# Conservation implications of genetic structure in the narrowest endemic quillwort from the Eastern Amazon

**DOI:** 10.1002/ece3.7812

**Published:** 2021-07-13

**Authors:** Jeronymo Dalapicolla, Ronnie Alves, Rodolfo Jaffé, Santelmo Vasconcelos, Eder Soares Pires, Gisele Lopes Nunes, Jovani Bernardino de Souza Pereira, José Tasso F. Guimarães, Mariana C. Dias, Taís Nogueira Fernandes, Daniela Scherer, Fernando Marino Gomes dos Santos, Alexandre Castilho, Mirella Pupo Santos, Emiliano Nicolas Calderón, Rodrigo Lemes Martins, Rodrigo Nunes da Fonseca, Francisco de Assis Esteves, Cecílio Frois Caldeira, Guilherme Oliveira

**Affiliations:** ^1^ Instituto Tecnológico Vale Belém Brazil; ^2^ Exponent Bellevue WA USA; ^3^ Instituto de Botânica de São Paulo São Paulo Brazil; ^4^ Programa Interunidades de Pós‐Graduação em Bioinformática Universidade Federal de Minas Gerais Belo Horizonte Brazil; ^5^ VALE S/A Gerência de Estudos Ambientais Licenciamento e Espeleologia Nova Lima Brazil; ^6^ Instituto de Biodiversidade e Sustentabilidade NUPEM Universidade Federal do Rio de Janeiro Macaé Brazil

**Keywords:** conservation genetics, effective population size, genomic skimming, isoetaceae, narrow endemic species (NES)

## Abstract

The quillwort *Isoëtes cangae* is a critically endangered species occurring in a single lake in Serra dos Carajás, Eastern Amazon. Low genetic diversity and small effective population sizes (*N*_e_) are expected for narrow endemic species (NES). Conservation biology studies centered in a single species show some limitations, but they are still useful considering the limited time and resources available for protection of species at risk of extinction. Here, we evaluated the genetic diversity, population structure, *N*_e_, and minimum viable population (MVP) of *I*. *cangae* to provide information for effective conservation programs. Our analyses were based on 55 individuals collected from the Amendoim Lake and 35,638 neutral SNPs. Our results indicated a single panmictic population, moderate levels of genetic diversity, and *N*_e_ in the order of thousands, contrasting the expected for NES. Negative F_IS_ values were also found, suggesting that *I*. *cangae* is not under risk of inbreeding depression. Our findings imply that *I*. *cangae* contains enough genetic diversity to ensure evolutionary potential and that all individuals should be treated as one demographic unit. These results provide essential information to optimize ex situ conservation efforts and genetic diversity monitoring, which are currently applied to guide *I*. *cangae* conservation plans.

## Graphical abstract

The quillwort Isoëtes cangae is a critically endangered species occurring in a single lake in Serra dos Carajás, Eastern Amazon. To provide information for effective conservation programs, we evaluated its genetic diversity and population structure using neutral SNPs. Our results indicated a single panmictic population, moderate levels of genetic diversity, and effective population size in the order of thousands, contrasting to the expected for NES.

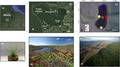

## 1 INTRODUCTION

Information on the genetic diversity and population structure can be used to support monitoring and conservation programs for threatened species, such as choosing priority populations for conservation (Fallon, 2007). Genetic information is even more valuable for endemic species with restricted distributions (Hamrick et al., 1991), known as “narrow endemic species” (NES), which are composed of one or a few populations limited to a specific habitat and confined to a small geographic area (Kruckeberg & Rabinowitz, 1985). A classical assumption about NES is their lower genetic diversity in comparison with widespread species due to their small effective population sizes (Gibson et al., 2008; Leimu et al., 2006; Smith & Pham, 1996). However, recent studies have shown that most Mediterranean plants considered as NES show moderate to high levels of genetic diversity (Fernández‐Mazuecos, 2014; Forrest et al., 2017; Jiménez‐Mejías, 2015; López‐Pujol et al., 2013). Such genetic data for NES from tropical areas such as Eastern Amazon are still scarce.

**FIGURE 1:**
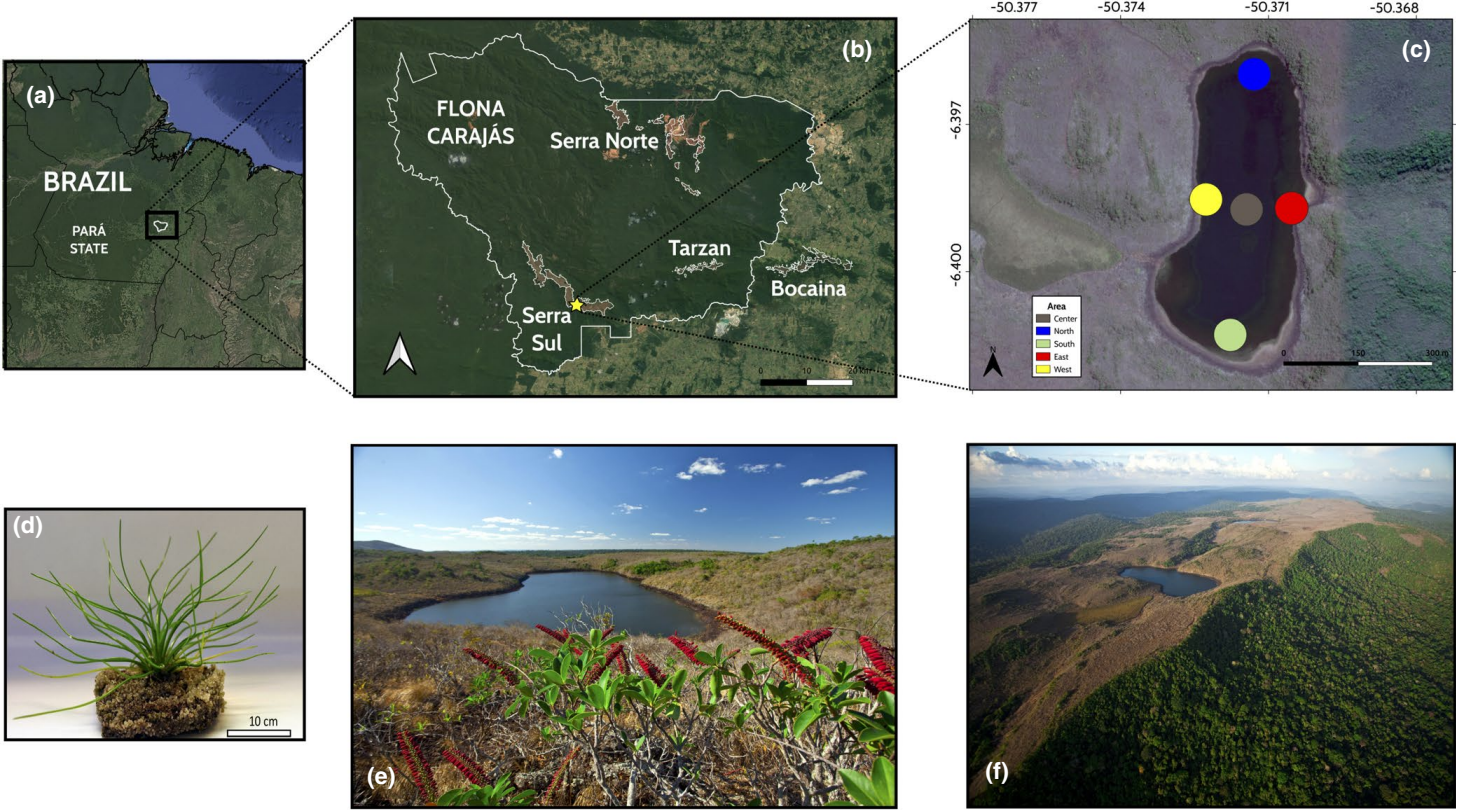
Map showing Serra dos Carajás, southeast Pará, Brazil (a), where is located the federal conservation unit Floresta Nacional de Carajás (FLONA de Carajás; thicker white border) and the four cangas areas (narrower white borders): Serra Norte, Serra Sul, Tarzan, and Bocaina (b). Amendoim Lake in the Serra Sul with five sampled areas (center, north, south, east, and west) of *Isoetes cangae* (c). Individual of *Isoetes cangae* sampled from Amendoim Lake. Photo: C. F. Caldeira (d). Landscape associated with the Amendoim Lake in the Floresta Nacional de Carajás, highlighting the open vegetation (e) and the ferruginous outcrop canga from Serra Sul (f). Photo: Cinthia M. Bandeira

Although the Amazon basin is usually represented as a predominant forest formation, there are several restricted and sparsely distributed open habitats within this biome, such as savannas, *campinaranas*, *cangas*, and *campos rupestres* (Devecchi et al., 2020; Pires & Prance, 1985). Among them, the *cangas* present one of the highest levels of diversity and endemism (Zappi et al., 2019), occurring among other areas, in the elevated plateaus of the mountain range of Serra dos Carajás, southeast of the state of Pará, Brazil, in the Eastern Amazon (Figure 1a–b). This mountainous complex is characterized by high iron ore concentrations under industrial exploration, thus requiring the establishment of species conservation plans (Freitas, 1986; Santos, 1986; STCP, 2016). Mining activities in the Serra do Carajás have been accompanied by scientific expeditions and botanical investigations (Viana et al., 2016). Thus, studies on the plant diversity from this mountain range started with many records of endemic genera and species (Giulietti et al., 2019). Among them, there are two heterosporous lycopods species of the genus *Isoëtes* L. that fit into NES definition: *I*. *cangae* J.B.S. Pereira, Salino & Stützel, and *I*. *serracarajensis* J.B.S. Pereira, Salino & Stützel (Isoetaceae; Pereira et al., 2016).

*Isoëtes* (popularly known as "quillworts" or "Merlin's grass") is a cosmopolitan genus of lycophytes with about 250 species found in lakes, wetlands (swamps, marshes), and terrestrial habitats (Troia, 2016a). The group is the only extant genus of the Isoetaceae and the single representative of Isoetales, which is a lineage of vascular plants that diverged from its living sister group *Selaginella* Willk in the Devonian (Pigg, 2001), with the diversification of extant species occurring in the Cenozoic (Wood, 2020). This lycopod lineage's ancient origin and its morphology make this genus a key group to understand vascular plants' evolutionary pathways (Hetherington et al., 2016). Species of *Isoëtes* show both dispersion capacities (hydrochory by single spores, sporangium, or whole sporophyll) and reproductive cycles dependent on water (Gentili et al., 2010; Troia, 2016). Thus, human activities leading to water quality deterioration, habitat loss, agriculture land‐use, and invasion by exotic species have increased the number of *Isoëtes* species listed as threatened worldwide (Chen, 2005, 2007; Gentili et al., 2010; Kang et al., 2005; Kim et al., 2008). Genetic studies in *Isoëtes* species have employed different approaches to characterize genetic diversity levels within and among populations, such as allozymes (Caplen & Werth, 2000; Chen et al., 2009; Small & Hickey, 1997), Amplified Fragment Length Polymorphism (AFLP; Jung et al., 2014; Kang et al., 2005), Inter‐Simple Single Repeat (ISSR; Gentili et al., 2010; Ma et al., 2019; Santos et al., 2020), microsatellites (Li et al., 2013; Stelt et al., 2017; Zheng et al., 2020), chloroplast DNA (Jung et al., 2014; Nunes et al., 2018; Zheng et al., 2020), or Random Amplified Polymorphic DNA (RAPD; Chen et al., 2007; Kim et al., 2008; Gentili et al., 2010). These studies have shown that some *Isoëtes* species are marked by low genetic diversity (Kang et al., 2005; Kim et al., 2008)⁠ and others by moderate and higher diversity (Gentili et al., 2010; Chen et al., 2012; Stelt et al., 2017). However, regardless of the genetic diversity index, several *Isoëtes* species are undergoing population decline (Kang et al., 2005; Gentili et al., 2010; Troia, 2016a; Stelt et al., 2017).

South America is one of the centers of diversity of the genus, and Brazil comprises 26 species, most of them considered as NES (Pereira et al., 2016; Flora do Brasil, 2020). However, specific conservation precautions need to be considered for *I*. *cangae*, which occurs in a single locality, the Amendoim Lake, submerged in the permanent and ultraoligotrophic lake (Figure 1c) in a ferruginous altitude field in the Serra Sul dos Carajás (Figure 1e–f) (Pereira et al., 2016; Viana et al., 2016). No modern nor historical palynological data of *I*. *cangae* were recorded in other lakes in the region (Absy et al., 2014; Guimarães et al., 2014, Guimarães et al., 2017; E. F. Silva, Lopes et al., 2020), indicating that this species is historically restricted to the Amendoim Lake. Its reduced area of occupancy (AOO) and extent of occurrence (EOO) have led to *I*. *cangae* being inserted in the red list of IUCN as a critically endangered (CR) species (Lansdown, 2019). Also, habitat quality deterioration due to intensive landscape alterations in the surrounding area, such as mining activities and forest conversion into pasturelands (Souza‐Filho et al., 2016), may bring future impacts on the species.

In the only population genetics study on *I*. *cangae,* Santos et al. (2020)⁠ found a high genetic diversity using ISSR markers with high gene flow among the sampling areas within the Amendoim Lake. However, the authors did not test the genetic structure using assignment tests nor estimated effective population size for the species, which are essential parameters to outline future conservation actions (Hoban et al., 2020). Furthermore, comparing genetic diversity levels using different markers allows access to new polymorphisms in new portions of the genome which can decrease the relative effects of gene flow and genetic drift in the observed genetic structure patterns (Freville et al., 2001). In addition, thousands of markers such as SNPs can bring more information about evolutionary processes, and more accurate estimates of demographic parameters, fundamental to optimize conservation biology efforts (Morin et al., 2009; Helyar et al., 2011; Torkamaneh et al., 2018). Few studies on *Isoëtes* applied high‐throughput sequencing technology to acquire genomic data, most of them were focusing on phylogenomics (Wood et al., 2020), phylogeography (Wood et al., 2018), local adaptation (Yang & Liu, 2016), or species delimitation (Nunes et al., 2018).

Conservation biology studies centered in a single species show some limitations (Simberloff, 1998)⁠ but they are still useful considering the limited time and resources available (Olden, 2003), and they are important either as models for management guidelines (Bichet et al., 2016)⁠ or for use of a single species as a focal, umbrella, or charismatic species (Watson et al., 2001; Pease et al., 2021; Politni et al., 2021). Here, we used SNPs from genomic data to estimate genetic diversity and population structure of the most endangered species of quillworts from Serra dos Carajás, *I*. *cangae*. This information will enhance genetic management capability both in situ and ex situ (Caldeira et al., 2019), aiming the conservation of this narrowly endemic species. We investigated how genetic diversity is structured in the Amendoim Lake, testing whether there are subpopulations and different management units for conservation strategies. We also estimated effective population size (*N*_e_) using genomic data to provide information about minimum viable population (MVP), a primordial data for species long‐term survival. Furthermore, with thousands of SNPs, we evaluated whether *I*. *cangae* is a Neotropical NES with low genetic diversity as classical studies supposed or, according to Santos et al. (2020)⁠ using ISSR markers, whether the species is a NES with high genetic diversity.

## 2 MATERIAL AND METHODS

### 2.1 Sampling effort

The Amendoim Lake is located in the Carajás National Forest (Floresta Nacional de Carajás; FLONA Carajás). The FLONA Carajás was created as a protected area for sustainable use, conciliating conservation, and mining activities of the ferruginous mountain outcrops from Serra dos Carajás. It sits 720 m above the sea level with an area of 1.23 km^2^ (Silva et al., 2018). The lake is hydrologically charged essentially by rainfall of a limited catchment basin. The water varies seasonally from ultraoligotrophic to oligotrophic conditions (Sahoo et al., 2017). The Amendoim Lake is found in the canga (Figure 1e–f) which is composed of several phytophysiognomies, comprising grasslands, scrublands, wetlands, and forest formations (Mota et al., 2015), differing in terms of the plant communities they support as well as in their soil chemistry (Mitre et al., 2018). The severe environmental conditions such as high temperature, UV radiation, high water loss, and poorly developed soils rich in metals are also peculiar of the canga (Jacobi et al., 2007), harboring endemics, and rare species (Giulietti et al., 2019).

We collected leaf samples of 55 specimens of *I*. *cangae* distributed along the whole area of the Amendoim Lake (Table S1) in five sampling areas, four of them representing similar areas used by Santos et al. (2020)⁠: north (*n* = 13), south (*n* = 10), east (*n* = 11), and west (*n* = 8), besides a fifth group to cover extra individuals located more centrally in the lake (center; *n* = 13) as shown in the Figure 1c. Samples were collected with a minimum distance of 2 m between plants to reduce the pool's relatedness. The collected plants were shipped in water to the laboratory. ICMBio/MMA granted sample collection under permits numbers 641,873, 64,403‐1, and 59,724. Voucher specimens were deposited at the RFA Herbarium, Universidade Federal do Rio de Janeiro (RFA‐UFRJ).

### 2.2 Library preparation

**FIGURE 2:**
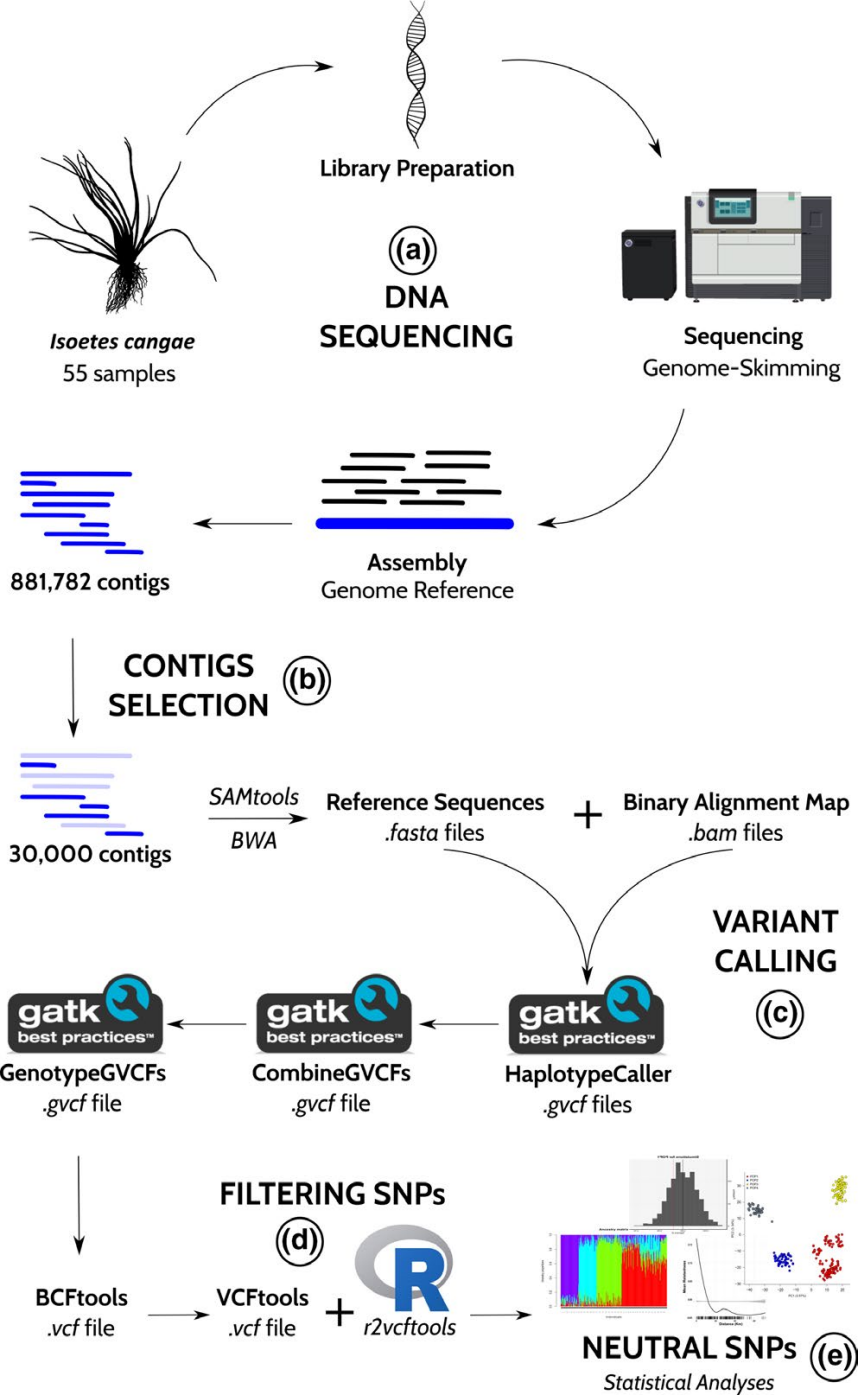
Methodological approach for sequencing and data analyses. (a) Genomic DNA sequencing was performed using one genomic library with 55 samples of *Isoetes cangae* from Amendoim Lake in the Serra dos Carajás, Pará, Brazil. The library was sequenced using the genome skimming method, and the assembly step was carried out using a reference genome. (b) We recovered 881,782 contigs, and we selected 30,000 contigs for the variant calling step (c). After filtering SNPs (d) by quality, we maintained only neutral SNPs for statistical analyses (e)

The genetic diversity analysis of *I*. *cangae* individuals was performed using the genome skimming approach (Figure 2a) (Malé et al., 2014; Weitemier et al., 2014; Wessinger et al., 2018). Shotgun paired‐end libraries were constructed from 50 ng of isolated DNA. For that, samples were subjected to a random enzymatic fragmentation in which the DNA was simultaneously fragmented and bound to adapters using the QXT SureSelect kit (Agilent Technologies). The fragmented DNA was purified using AmPure XP beads (Beckman Coulter) and subjected to an amplification reaction using primers complementary to the Illumina flowcell adapters. Amplified libraries were again purified using AmPure XP beads (Beckman Coulter), quantified using the Qubit 3.0 Fluorometer (Thermo Fisher Scientific Inc.), and checked for fragments size in the 4,200 TapeStation (Agilent Technologies®) using a ScreenTape DNA 1,000 kit (Agilent Technologies). The libraries were adjusted to a 4 nM concentration, pooled, denatured, and diluted to a running concentration of 1.8 pM. The sequencing run was performed in the NextSeq 500 Illumina platform using a NextSeq 500 v2 kit high output (300 cycles).

### 2.3 Reference genome and contigs selection

A draft genome of *I*. *cangae* (ITV2008_illumina) was assembled using a total of 4.3 Gb paired‐end reads (28.595.407 pared‐end reads totaling 8.603.589.014 bases, with an average length of 150 bp) sequenced by NextSeq 500 Illumina platform. The draft de novo genome assemblies were generated using SPAdes v3.10.0 (Bankevich et al., 2012)⁠ with the following parameters (‐‐careful ‐‐only‐assembler ‐k 21,33,55,77,99,127). The draft genome "ITV2008_illumina" was adopted as a reference for variant analysis with 55 samples of *I*. *cangae*. We selected 30,000 contigs at random with a minimum size of 100 bp (Figure 2b) from the draft genome, namely "icangae_ref30k," to produce the reference files at an individual level. For each sample, the alignment was made with the reference "icangae_ref30k" using the programs SAMtools v1.19 (Li et al., 2009)⁠ and Burrows‐Wheeler Aligner (BWA) v7.17 (Li & Durbin, 2010).

### 2.4 Variant calling and SNP filtering

We employed the 30,000 contigs to align and build three files for each sample: (i) reference sequences file (.*fasta* files) in SAMtools; (ii) binary alignment map (.*bam* files); and (iii) sequence dictionary file (.*dict* files) in Picard v.2.22.9 (http://broadinstitute.github.io/picard/). All these files were used to perform the variant calling step (Figure 2c) in GATK v.4.1.5.0 (McKenna et al., 2010). First, we created.*gvcf* files for each sample using the *HaplotypeCaller* function (Poplin et al., 2017). Then, we combined the resulting files into a single.*gvcf* by applying the function *CombineGVCFs*. Next, the genotype calls were verified and corrected with the *GenotypeGVCFs* function to improve the genetic mapping accuracy. Finally, we used BCFTools v1.10.2 (http://www.htslib.org/) to convert the final.*gvcf* into a.*vcf* file.

We filtered SNPs from the final.*vcf* file (Figure 2d) with VCFtools v0.1.16 (Danecek et al., 2011)⁠ and the "r2vcftools" package (Pope, 2020)⁠ for the R package v3.6.3 (R Core Team, 2020). We excluded indels and maintained only biallelic SNPs as *I. cangae* is a diploid species (data not shown). Afterward, we selected SNPs by quality, keeping only SNPs with (i) with 10% of maximum missing data value because a large amount of missing data may affect some demographic parameters (Marandel et al., 2020)⁠ and analyses (Novembre & Stephens, 2008; Helyar et al., 2011); (ii) with an allelic frequency greater than 5%; and (iii) a depth coverage range between 50 and 200 reads. We then filtered our dataset by Linkage Disequilibrium, allowing SNPs with a maximum correlation of 40% (*R²* < .4; Xuereb et al., 2018), and excluded strong deviations from the Hardy–Weinberg equilibrium (HWE, *p* > .0001; O’Leary et al., 2018). We also removed potential SNPs under selection, through genome scans, using F*_ST_* outlier tests in *snmf* function in the "LEA" package (Frichot & Francois, 2014). We corrected for false discovery rates, using the genomic inflation factor and calibrated *p*‐values with the Benjamini–Hochberg algorithm (Q = 0.05) (François et al., 2016). Thus, we employed only neutral SNPs for subsequent analyses (Figure 2e).

### 2.5 Genetic structure

Neutral SNPs were used to estimate population structure with two approaches: sparse Nonnegative Matrix Factorization (sNMF) and Discriminant Analysis of Principal Components (DAPC). Both methods are model‐free, and there are no assumptions regarding the population model, unlike other clustering methods (Fenderson et al., 2020). sNMF was performed in "LEA" to estimate the individual ancestry coefficients, allowing the inference of the number of ancestral populations (*K*) that would correspond to the groups or genetic clusters (Frichot et al., 2014). We used the "adegenet" R package (Jombart & Ahmed, 2011)⁠ to carry out the DAPC and to investigate the probability of individuals belonging to each genetic cluster observed, applying a Discriminant Function Analysis (DFA) with Principal Components (PC) (Jombart et al., 2010). This approach reduced the data dimensionality without losing genetic information and returning the best number of genetic clusters to explain the current genetic structure (Jombart et al., 2010).

For sNMF and DAPC, we tested *K* values between 1 and 10. In sNMF, different values for the regularization parameter (α) were tested to evaluate possible changes in the best K value (Frichot et al., 2014), with 10 replications for each value (αvariance to verify changes in the estimated = 10, 100, 500, 1,000, 2,000, 4,000). Plots were constructed to visualize the lower cross‐entropy value by *K* in each α. The number of *K* with the lowest cross‐entropy represented the number of ancestry populations. In DAPC, all PCs (100% of the variance) were used to select the best *K* using the Bayesian information criterion (BIC) in the function *find.clusters* (Jombart et al., 2010). We also tested the *find.clusters* function with other numbers of PCs, representing 95%, 75%, and 50% of the variance to verify changes in the estimated *K* (Miller et al., 2020). BIC was interpreted like cross‐entropy: the lower its value, the more likely this *K* value represents the number of genetic clusters. Finally, we ran a principal components analysis (PCA) with "adegenet" to plot sample genotypes in the multivariate space according to the five sampled areas and thus visually assess any potential clustering following a spatial pattern (Jombart et al., 2009).

### 2.6 Genetic diversity, genetic distance, and effective population size

Genetic diversity indexes and their confidence intervals (C.I. 95%) were also calculated for *I*. *cangae*, employing the neutral SNPs and *Query* function using "r2vcftools." We estimated the observed heterozygosity (He_obs_), expected heterozygosity (He_exp_), inbreeding coefficient (*F*_IS_) at the individual and population levels, and nucleotide diversity (π) only for population level. Significance of different diversity values (among individuals in different sampling areas) was assessed with Tukey's test using *Query* function as well. Also, the Yang's Relatedness coefficient (Rel; Yang et al., 2010)⁠ considered an indirect measure of recent gene flow (Carvalho et al., 2019)⁠ was estimated using the *Relatedness* function in "r2vcftools." Relatedness indexes show the genome proportion between two individuals that are identical by descent (Lynch & Ritland, 1999). Relatedness values greater than 0.25 indicate that the pair of individuals are related. In contrast, values around zero represent unrelated individuals as expected in a population under panmictic conditions. Large negative values mean more unrelated individuals than expected under panmixia, indicating genetic structure or barriers (Norman et al., 2017).

We estimated effective population size (*N*_e_) and its confidence interval (C.I. 95%) using the linkage disequilibrium (LD) method (Waples & Do, 2008)⁠ in NeEstimator v.2.1 (Do et al., 2014). Since a large number of SNPs and missing values (Waples & Do, 2010; Marandel et al., 2020)⁠ could bias the N_e_ estimates when using the LD method, we resampled the final dataset (neutral SNPs), without replacement and missing values, producing datasets with different number of SNPs, between 5,000 and 30,000. We calculated N_e_ for all the resampled datasets to assess the sensitivity of our estimates to missing SNPs.

To calculate the minimum viable population (MVP) for *I. cangae*, we applied two formulas: MVP_50_ = (50 × *N*_c_)/*N*_e_ considering 50 as the minimum population size necessary to avoid inbreeding problems, and MVP_500_ = (500 × *N*_c_)/*N*_e_ using 500 as the minimum population size to maintain species evolutionary potential (Jamieson & Allendorf, 2012). We employed the *N*_e_ value—and confidence interval—found in the complete dataset with all neutral SNPs for MVP estimates. *N*_c_ indicates the census size of the species (representing the total number of individuals) and was estimated through a survey according to Santos et al. (2020) by free‐diving in the perimeter of the Amendoim Lake up to the 4 m isobath in October 2018—in deeper areas *I*. *cangae* was not observed. We sampled 190 points randomly distributed, but in order to understand the entire perimeter of the lake. In each point, a 1 × 1 m square subdivided into four identical quadrants was placed at the bottom. The coverage of *I. cangae* in the square was estimated visually by the diver. To establish the relationship between density and coverage of *I*. *cangae*, the number of specimens was also counted in 20 of the sampled points selected at random within four coverage categories (0%–24%, 25%–49%, 50%–74%, 75%–100%). Densities were correlated with estimated coverage visually to establish a mathematical relationship between the coverage and the density. We applied this correlation to estimate the density of specimens in the remaining 170 squares sampled. Not all specimens were directly counted in all squares due to the limited time and divers available to carry out the sampling. *N*_c_ for all lake up to a depth of 4 m was calculated by multiplying the average estimated density of specimens (specimens of *I*. *cangae* per m²) with the size of the sampled area (in m²). To measure the size of the sampled area, the Google Earth Pro geometric area measurement tool (Google LLC. V7.3.3.7796) was used, using the satellite image and the coordinates of the sampled points to define the limits of the geometric area measured. All R scripts for the methodological steps are available in https://github.com/jdalapicolla/Isoetes_Scripts.R.

## 3 RESULTS

### 3.1 Neutral SNPs

The draft genome adopted as a reference, namely "ITV2008_illumina," contained 881,782 contigs (N50: 1,287; L50: 59,243; total base pairs (bp): 268,606,676). From this reference, "icangae_ref30k" (N50: 6,742; L50: 555; total (bp): 16,698,786) was selected with 30,000 contigs (Table S2). Each sample resulted in approximately seven million reads, with a total of 810,000,051 bp, and most contigs showed fragment sizes between 250 and 500 bp (Table S2).

The variant call step identified 2,349,431 variants in the.*vcf* file for 55 individuals, which after filtering by quality, were reduced to 71,621 SNPs (Table S3). Later, we selected 35,638 neutral SNPs (Figure S1) for our final dataset used in the subsequent analyses. The final dataset showed a mean coverage depth of 83.8 reads by SNP (Median = 71.49), with 9.09% and 0.40% of the maximum amount of missing data per locus and individuals, respectively (Table S1).

### 3.2 Genetic structure

**FIGURE 3:**
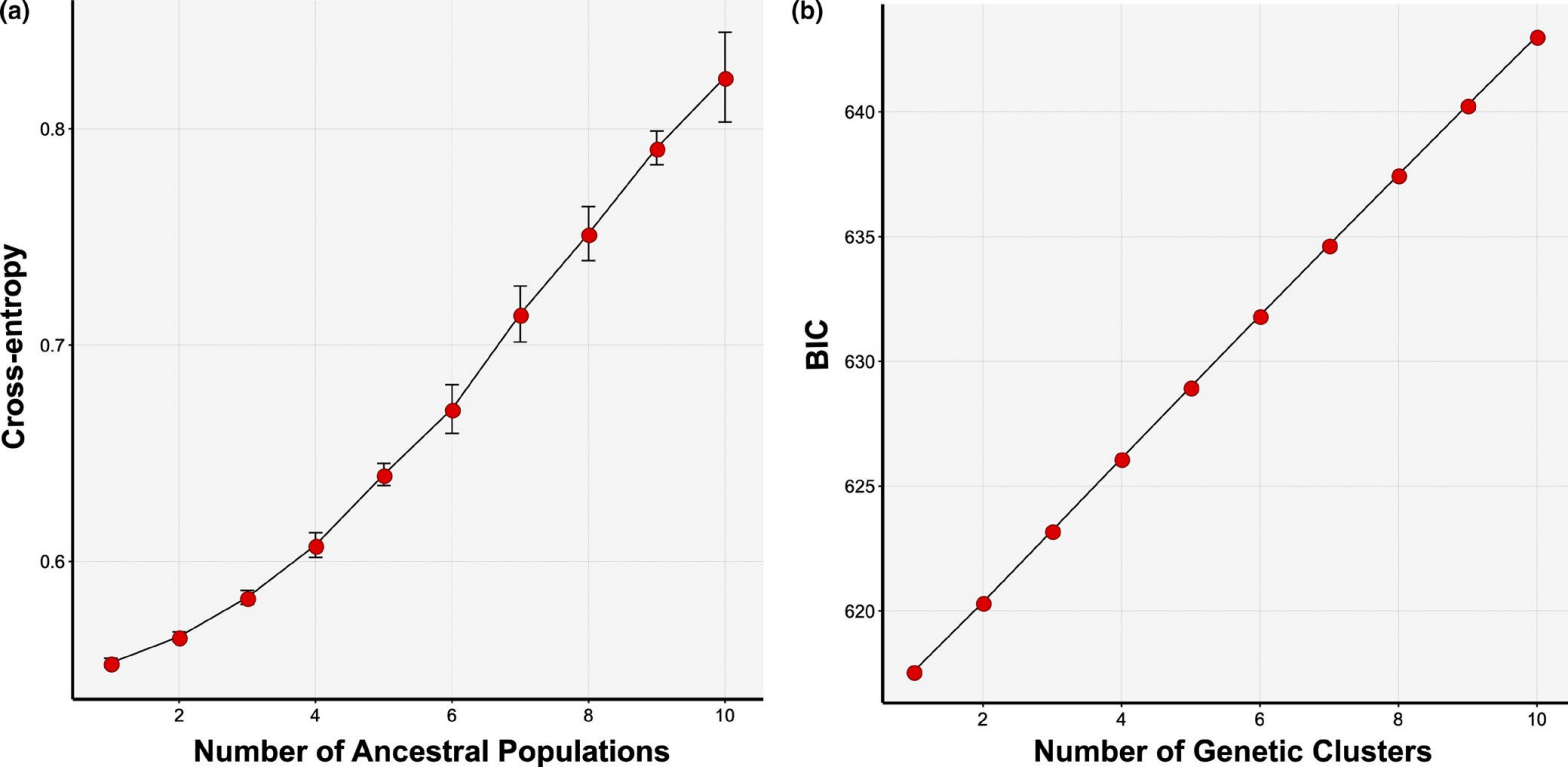
Number of ancestral populations (K) of *Isoetes cangae* estimated using the sparse Nonnegative Matrix Factorization (sNMF) with α = 1,000, cross‐entropy values, and error bars for 10 replicates (a). The number of genetic clusters (K) estimated employing Discriminant Analysis of Principal Components (DAPC) with all PCs and BIC values (b). Regardless of the regularization parameter α values in sNMF and the number of PCs in DAPC, the number of clusters with the lowest cross‐entropy and BIC values was 1 (Figure S2–S3)

**FIGURE 4:**
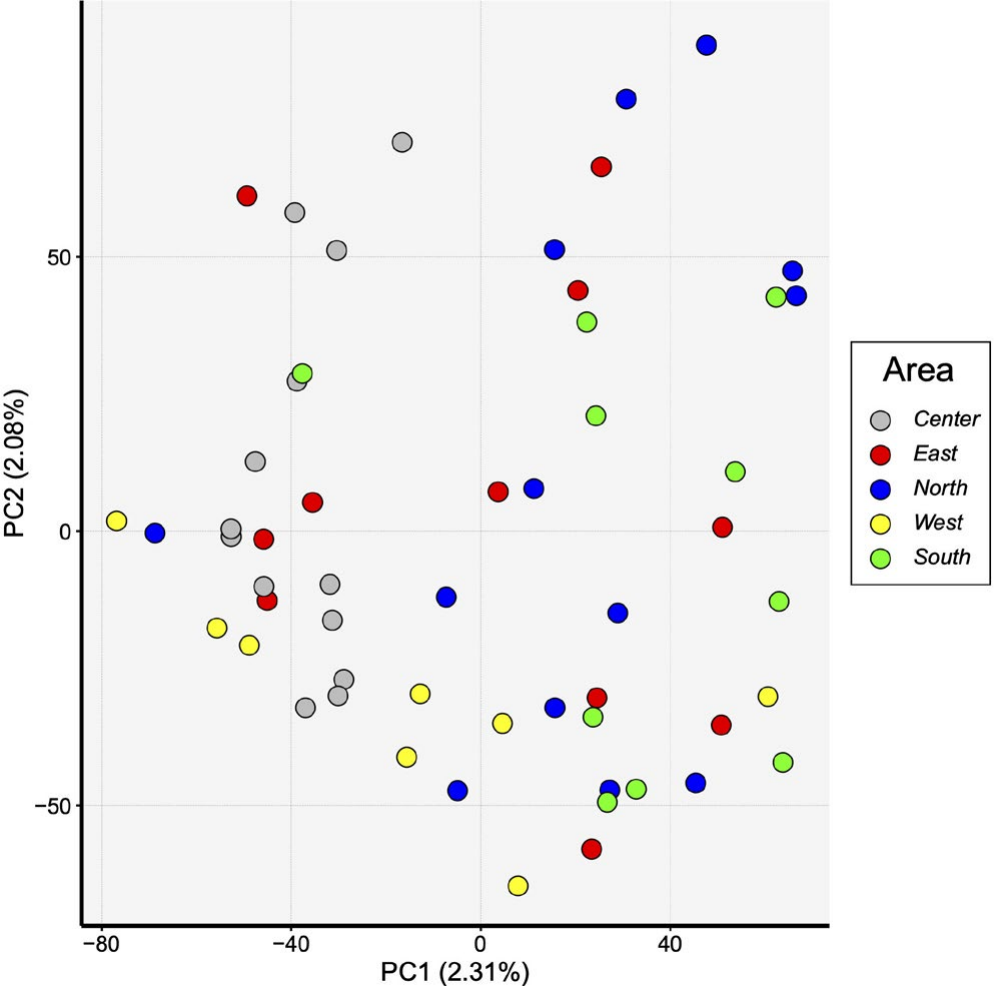
First two principal components (PC) for the principal component analysis (PCA) with all individuals of *Isoetes cangae*. The colors represent the five sampled areas at Amendoim Lake in the Floresta Nacional de Carajás

Both approaches for estimating genetic structure, sNMF, and DAPC, recovered one single cluster in *I. cangae* (Figure 3). In sNMF (Figure 3a), the lowest value of cross‐entropy was 0.549 for α = 1,000 and all α values showed one ancestral population K = 1 (Figure S2). The best value for DAPC (BIC =617.6) was found when K = 1 (Figure 3b), and the number of PCs did not affect the results (Figure S3). Also, PCA did not indicate multiple clusters (Figure 4), corroborating the sNMF and DAPC results.

### 3.3 Genetic diversity, genetic distance, and effective population size

**FIGURE 5:**
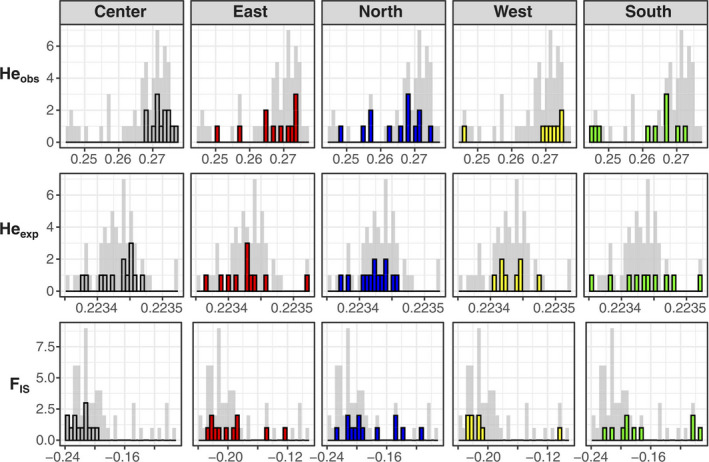
Frequencies of observed values in the three genetic diversity metrics observed heterozygosity (He_obs_), expected heterozygosity (He_exp_), and inbreeding coefficient (*F*_IS_) at the individual level. Gray bars represent all 55 individuals, and the highlighted and colored bars represent the frequencies in each sampling area by column. Center area highest values for He_obs_ and lowest for *F*_IS_ while in the other sampling areas the values are more distributed

Nucleotide diversity in *I. cangae* was 0.224, and the species showed a high heterozygosity level (He_obs_ > He_exp_). The observed heterozygosity of 0.267 and the expected heterozygosity of 0.224 lead to a negative inbreeding coefficient value (*F*_IS_ = −0.194) (Table 1). Considering the genetic diversity indices at the individual level, only the individuals from the center and west of the Amendoim Lake showed concentrated higher values for He_obs_ and lower for *F*_IS_ (Figure 5). However, Tukey's tests only showed significant differences for He_obs_ in all comparisons including west area and in all comparison regarding the area for He_exp_ (Figure S4). We performed 1,485 comparisons between individuals for Yang's relatedness coefficient (Rel), which returned negatives values close to zero [mean = −0.016; standard deviation (*SD*) = 0.005], indicating panmictic conditions (Figure S5).

**TABLE 1:** Genetic diversity indexes with their 95% confidence intervals (C.I.) for 55 individuals of *Isoetes cangae* from Amendoim Lake, Serra dos Carajás, Pará, Brazil

	Mean	C.I. (95%)
He_obs_	0.267	0.265 – 0.269
He_exp_	0.224	0.223 – 0.224
*F*_IS_	−0.194	−0.204 – −0.184
Π	0.224	0.222 – 0.225

Abbreviations: *F*_IS_, inbreeding coefficient; He_exp_, expected heterozygosity; He_obs_, observed heterozygosity; π, nucleotide diversity.

N_e_ estimates using different SNP numbers showed discrepancies in the results with mean values ranging from 24,303.4 to 124,424.3 (Table S4). *N*_e_ estimates based on all 35,638 neutral SNPs recovered 64,226.2 (C.I 95% = 45,557.2 – 108,852.2) individuals (Table S4). *N*_c_ was estimated around 200,000 *I*. *cangae* individuals for the Amendoim Lake (C.I. 95% = 160,000 – 240,000), and considering the minimum population size as 50 (MVP 50), the number of individuals necessary to the long‐term viability of the only population of *I*. *cangae* was between 92 and 220. Considering the minimum population size as 500 (MVP 500), a population 10 times larger would be necessary with the number of individuals ranging between 919 and 2,195 (Table 2).

**TABLE 2:** Estimates for minimum viable population (MVP) in *Isoetes cangae* according to two criteria of *N*_e_ following Jamieson and Allendorf (2012): MVP_50_ and MVP_500_. We applied the *N*_e_ value calculated using all neutral SNPs in the complete dataset and its 95% confidence interval (Table S4). For census number (*N*_c_), we applied in all criteria the mean number of individuals found in the survey performed in Amendoim Lake

95% Confidence Interval	*N*_e_	*N*_c_	MVP_50_	MVP_500_
Lower	45,557	200,000	220	2,195
Mean	64,226	200,000	156	1,557
Upper	108,853	200,000	92	919

Abbreviations: MVP, Minimum viable population; *N*_c_, Census number; *N*_e_, Effective population size.

## 4 DISCUSSION

Here, we addressed the population genomics of an endangered Neotropical NES of *Isoëtes*, applying SNPs to provide information for management and conservation programs. We showed that *I*. *cangae* is composed of a single panmictic population with moderate genetic diversity and no inbreeding signal, contradicting the classical assumptions for a NES. Population genetics studies of *Isoëtes* species around the world have been reported different levels of genetic diversity in *Isoëtes* species, applying other genetic markers (Caplen & Werth, 2000; Chen et al., 2005; Kang et al., 2005; Kim et al., 2008; Gentili et al., 2010; Li et al., 2013; Stelt et al., 2017; Ma et al., 2019; Zheng et al., 2020). Santos et al. (2020)⁠ also found a high genetic diversity using ISSR markers but the presence of a genetically differentiated on the North of the Amendoim Lake was not corroborated by our results. Genetic diversity metrics at the individual level showed that the expected heterozygosity is significantly different in each area but the observed heterozygosity is higher and significantly different in the west sampling area, indicating that this area holds more genetic diversity. However, these differences among areas are too small (see the scale of the axis in Figure 5 and Figure S4), not being enough to recover any subpopulation in the assignment population tests. Individual inbreeding coefficient did not differ between areas, suggesting there is no risk of inbreeding among individuals of *I*. *cangae*.

These differences can be attributed to our sampling effort, employing more individuals, and to thousands of markers (SNPs) used in analyses which allowed us a broader explanatory power on neutral genetic structure. SNPs are codominant markers that may represent different portions of the genome. SNPs contain enough information for population genetics analyses while providing a refined and accurate genomic data source to access the genetic structure and diversity at a low cost (Allendorf et al., 2010; Helyar et al., 2011; Angeloni et al., 2012). Previous studies have been successfully using this approach to study other endemic and endangered species of angiosperms from the *cangas* in Serra dos Carajás (e.g., Lanes et al., 2018; Carvalho et al., 2019), besides the analyses conducted with several other groups of vascular plants from distinct regions in the planet (e.g., Wickell et al., 2017; Wolf et al., 2019; Wang et al., 2020).

We followed Forrest et al. (2017)⁠ categorization for genetic diversity (He_exp_) that consists into three categories ranging between 0 and 0.5: low diversity from 0 to 0.166, moderate diversity from 0.167 to 0.333, and high diversity from 0.334 to 0.500. In this rank, *I*. *cangae* showed a moderate genetic diversity (He_exp_). The previous study using SNPs for an *Isoëtes* species (Wood et al., 2018)⁠ did not provide any information on the genetic or nucleotide diversity to enable a comparison with our results. Therefore, we compared genetic diversity indexes of *I*. *cangae* with those of other plant species, all angiosperms which employed SNPs in the analyses, being most of them NES in *cangas* in Serra dos Carajás as well. All these species fitted into moderate genetic diversity category. *Isoëtes cangae* showed greater genetic (He_exp_) and nucleotide (π) diversities than the annual herb *Monogereion carajensis* Barroso & King (He_exp_ = 0.16–0.18; Carvalho et al., 2019)⁠ and *Ipomoea cavalcantei* Austin (He_exp_ = 0.18; π = 0.18), an endangered NES from Serra dos Carajás (Lanes et al., 2018). On the other hand, *I*. *cangae* exhibited similar genetic diversity and greater π values than other abundant species in *cangas* such as *Ipomoea maurandioides* Meisn. (He_exp_ = 0.17–0.23; π = 0.13–0.15) (Lanes et al., 2018), and for the NES *Brasilianthus* *carajensis* Almeda & Michelang. (He_exp_ = 0.21–0.25; π = 0.16–0.21) (A. R. Silva, Resende‐Moreira et al., 2020). Finally. *I. cangae* showed smaller genetic diversity index than the NES *Mimosa acutistipula* var. *ferrea* Barneby (He_exp_ = 0.28; π = 0.23–0.26) and *Dioclea apurensis* Kunth (He_exp_ =0.25–0.29; π = 0.21–0.25), wider distributed species in the Amazon (Carvalho et al., 2021). Although other studies on *Isoëtes* have used different genetic markers, making our genetic indices not directly comparable, several of them also recovered moderate and high genetic diversity values, even for threatened species such as *I*. *hypsophila* Hand.‐Mazz. (Chen et al., 2005), *I*. *sinensis* Palmer (Kang et al., 2005), and *I*. *malinverniana* Ces. & De Not. (Gentili et al., 2010).

We found no evidence of inbreeding in *I*. *cangae*, as seen in other *Isoëtes* species (Stelt et al., 2017)⁠ despite showing a restricted distribution range without other populations to maintain gene flow. Rather, there is an indication of outbreeding with negative F_IS_ values. Small and Hickey (1997)⁠ also found a small and negative F_IS_ for *I*. *karstenii* Braun., a Neotropical species from the high‐altitude paramos of Merida, Venezuela. The authors interpreted these results as evidence of near‐random mating within subpopulations, without inbreeding or outbreeding. F_IS_ values provide a coefficient of “correlation between uniting gametes” (Wright, 1922, 1965). A high positive correlation will generate inbred offspring. In contrast, a low negative correlation will generate more heterozygous offspring (Johnson & Shaw, 2015). Usually, negative F_IS_ values resulting in more heterozygous individuals than expected might be explained by (i) reproductive mechanisms preventing inbreeding or enhancing the breeding of unrelated individuals or (ii) hybridization between different species or distant populations (Dobzhansky, 1950).

Regarding reproductive mechanisms, it is unclear whether *I*. *cangae* reproduces predominately sexually in its natural habitat. Caldeira et al. (2019)⁠ indicated that *I*. *cangae* individuals reproduced essentially sexually in controlled conditions and the maturation and release of both microspores and megaspores are synchronized with no evidence of self‐incompatibility (Caldeira et al., 2019; Santos et al., 2020). For sexual reproduction, the male gamete of *Isoëtes* present multi‐flagella distributed along more than two‐thirds of the cell length, resulting in a more efficient swim than other multiflagellate lycophytes and ferns (Renzaglia & Garbary, 2001). This efficiency would allow gametes to reach the different portions of the lake, and consequently, a wide crossbreeding capacity between individuals from different parts of the lake. Other factors could help gametes' dispersal capacity, such as the frequent wind on the water surface (Guimarães et al., 2014; Silva et al., 2018), creating currents connecting the opposite sides and leading to negative F_IS_ patterns in *I*. *cangae*. Stelt et al. (2017)⁠ indicated inbreeding in *I*. *butleri* Englem., an ephemeral wetlands species from North America, due to genetic structure and low dispersal ability. Therefore, dispersion and connection among individuals may be greater in a submerged species as *I*. *cangae* with its spores being transported all over the year (Caldeira et al., 2019; Santos et al., 2020), guiding to an opposite genetic pattern for other NES. Also, outbreeding can arise within a single population after generations of local adaptation in selfing plant species (Fischer & Matthies, 1997; Johnson & Shaw, 2015), even in small distances such as 30 m (Waser & Price, 1994). Subsequent crossings between inbred lines with different local adaptations can generate negative F_IS_ (Edmands, 2007; Johnson & Shaw, 2015).

Hybridization does not fit as the process responsible for this *F*_IS_ pattern. Although hybridization is a typical process in other *Isoëtes* species (Kim et al., 2010; Pereira et al., 2019), the only other species of the genus reported to the region (*I*. *serracarajensis*) is not sympatric in the same habitat, being found in temporary lakes, and intense fieldworks in the area never registered any hybrids between them (Nunes et al., 2018; Caldeira et al., 2019; Santos et al., 2020). In addition, historically *I*. *cangae* is only reported in the Amendoim Lake (Absy et al., 2014; Guimarães et al., 2014, Guimarães et al., 2017; E. F. Silva, Lopes et al., 2020). Gene flow between distant populations, creating contact areas and leading to outbreeding in *I*. *cangae*, is also unlikely because the only known population occurs in the Amendoim Lake (Pereira et al., 2016)⁠ and our analyses did not find a genetic structure.

Usually, conservation programs based on genetic data aim to reduce the loss of genetic diversity through genetic drift, increasing the number of individuals by translocation of adult individuals (Johnson et al., 2010), or transplantation via spores between populations (Hufford & Mazer, 2003). Our results indicated that *I*. *cangae* individuals should be treated as one demographic unit for conservation and management purposes. Furthermore, our estimates for the effective population size of *I*. *cangae* using the LD method showed a large population size even in lower bound of confidence intervals, *N*_e_ > 500, which probably would allow the species to adapt to environmental changes (Jamieson & Allendorf, 2012; Hoban et al., 2020). Population census estimates indicated the presence of 200,000 individuals in the Amendoim Lake, which is in accordance with our N_e_ evaluation.

MVP estimates can be interpreted as the minimum number of individuals that need to be rescued in an ex situ conservation strategy to maintain future generations' current genetic diversity in *I*. *cangae*. While Hoban et al. (2020)⁠ suggested using the MVP of 500 for genetic conservation studies and management, our results showed that 919 individuals are the minimum number of individuals for an ex situ conservation strategy in an optimistic scenario, with 2,195 individuals being recommended for a more conservative scenario. Although the MVP indicated a feasible number compared with the census number (200,000 individuals), this result must be analyzed with caution, since this estimate is not considering other population parameters, such as generation time, species recruitment rate, local adaptation, and the population persistence (Frankham et al., 2014). Even considering the caveats, our MVP estimation is a good starting point to a viable ex situ conservation strategy aiming for a minimum population that will not suffer from inbreeding in the short and medium term (Allendorf et al., 2010; Jamieson & Allendorf, 2012). Usually, there are concerns about inbreeding in endangered species (Frankham et al., 2017). Still, our results suggested the occurrence of outbreeding for *I*. *cangae*, which also needs to be considered in conservation programs for this species.

The vulnerability of *I*. *cangae* goes beyond being an NES, restricted to a single lake in Eastern Amazon. Habitat loss due to the mining activities and climate change may affect this species in the future (Santos, 1986; Souza‐Filho et al., 2016; STCP, 2016),⁠ and a lot of effort has been made to get information for the management of this species. Caldeira et al. (2019)⁠ developed protocols for ex situ propagation and growth for *I*. *cangae* that showed low sporeling mortality, suggesting that *I*. *cangae* is a resilient species able to grow and colonize environments with a broad temperature range and different types of substrates with low trophic characteristics. The development of viable spores for transplantation in the wild has already been suggested as a conservation strategy for *I*. *coreana* Chung & Choi (Kim et al., 2008), *I*. *sabatina* Troia & Azzella (Magrini et al., 2020), besides being successfully applied in the ex situ conservation of *I*. *malinverniana* (Abeli et al., 2018). Recently, evaluating the ecophysiology of *I*. *cangae*, Zandonadi et al. (2021)⁠ produced sporophytes ex situ under natural light which were successful when reintroduced in situ environment. Our findings provide genomic data and practical actions for the managements programs such as the minimal number of individuals (MVP) for an ex situ conservation approach, and the possibility of selecting individuals throughout the Amendoim Lake, since *I*. *cangae* comprises a single population. A random selection of individuals for propagation and other studies is possible even with significant differences in genetic diversity between the areas because these differences are small and we did not found differences between areas associated with *F*_IS_ values.

In short, our results showed that the only known population of *I*. *cangae* consists of a single panmictic population as initially hypothesized. As other NES from Mediterranean areas and from other species in *cangas*, *I*. *cangae* presented a moderate genetic diversity, with possible outbreeding instead of inbreeding. Heterozygous individuals are more common in west area of the lake but with little differences when compared to other areas. Higher heterogeneity might be better explained by reproductive mechanisms, such as multiflagellate male gametes, which enhance breeding of unrelated individuals rather than hybridization among different species or populations. Population size results showed that *I*. *cangae* is not under inbreeding depression, and it has evolutionary potential for long‐term survival. Also, conservation programs must pay attention to the risk of outbreeding depression in this species. The conservative number of around 2,200 individuals is needed to make ex situ conservation viable for long‐term programs. Currently, plants gathered from different portions of the Amendoim Lake are cultivated in controlled conditions providing reproductive structures to a large‐scale ex situ propagation. With more than 4,000 plants regenerated in vitro until now, such individuals represent (i) a significant genetic pool of *I*. *cangae* ensured ex situ and (ii) a support for several ongoing studies, including plant performance in field trials aiming to define more suitable habitat for translocation/reintroduction and optimize conservation efforts.

## CONFLICT OF INTEREST

None declared.

## AUTHOR CONTRIBUTIONS

**Jeronymo Dalapicolla:** Data curation (equal); Writing‐original draft (equal). **Ronnie Alves:** Data curation (equal); Formal analysis (equal); Validation (equal). **Rodolfo Jaffe:** Conceptualization (equal); Formal analysis (equal); Investigation (equal); Writing‐original draft (equal). **Santelmo Vasconcelos:** Conceptualization (equal); Data curation (equal); Writing‐review & editing (equal). **Eder Soares Pires:** Formal analysis (equal); Methodology (equal); Resources (equal). **Gisele Lopes Nunes:** Formal analysis (equal); Methodology (equal). **JOvani Bernardino de Souza Pereira:** Investigation (equal); Methodology (equal); Validation (equal). **José Tasso Felix Guimarães:** Formal analysis (equal); Methodology (equal). **Mariana Costa Dias:** Formal analysis (equal); Methodology (equal). **Tais Nogueira Fernandes:** Funding acquisition (equal); Project administration (equal); Validation (equal). **Daniela Scherer:** Funding acquisition (equal); Project administration (equal); Validation (equal). **Fernando Marino Gomes dos Santos:** Funding acquisition (equal); Project administration (equal); Validation (equal). **Alexandre Castilho:** Funding acquisition (equal); Project administration (equal); Validation (equal). **Mirella Pupo Santos:** Conceptualization (equal); Investigation (equal); Validation (equal); Visualization (equal). **Emiliano Nicolas Calderón:** Data curation (equal); Investigation (equal); Methodology (equal). **Rodrigo Lemes Martins:** Funding acquisition (equal); Project administration (equal); Validation (equal). **Rodrigo Nunes Fonseca:** Conceptualization (equal); Project administration (equal); Validation (equal). **Francisco de Assis Esteves:** Funding acquisition (equal); Project administration (equal); Validation (equal). **Cecilio F Caldeira** **:** Conceptualization (equal); Data curation (equal); Funding acquisition (equal); Investigation (equal); Project administration (equal); Writing‐review & editing (equal). **Guilherme Corrêa Oliveira:** Conceptualization (equal); Funding acquisition (equal); Investigation (equal); Project administration (equal); Validation (equal); Writing‐review & editing (equal).

### DATA AVAILABILITY STATEMENT

Draft genome for *Isoetes cangae*; R scripts for all analyses; raw VCF and Filtered VCF files; and the Supplementary Information are available in Dryad (https://datadryad.org/stash/share/3rWc5i‐8oYxy‐I_NCl44FHy8ElweMsztdvvv0e2_bv0).

## Supporting information

Supplementary Material
