# Supplementary material for: Conservation implications of genetic structure in the narrowest endemic quillwort from the Eastern Amazon
Source: Ecol Evol. 2021 Jul 13;11(15):10119–32. doi: 10.1002/ece3.7812 (PMC8328431; doi:10.1002/ece3.7812)
Supplement: Supplementary file 1 — Supplementary Material [file ECE3-11-10119-s001.docx]

**Supplementary Information**

Conservation implications of genetic structure in the narrowest endemic quillwort from the Eastern Amazon

Jeronymo Dalapicolla, Ronnie Alves, Rodolfo Jaffé, Santelmo Vasconcelos, Eder Soares Pires, Gisele Lopes Nunes, Jovani Bernardino de Souza Pereira, José Tasso F. Guimarães, Mariana C. Dias, Taís Nogueira Fernandes, Daniela Scherer, Fernando Marino Gomes dos Santos, Alexandre Castilho, Mirella Pupo Santos, Emiliano Nicolas Calderón, Rodrigo Lemes Martins, Rodrigo Nunes da Fonseca, Francisco de Assis Esteves, Cecílio Frois Caldeira, and Guilherme Oliveira.

**TABLES**

**Table S1:** Individuals of *Isoetes cangae* used in genomic analyses with geographical coordinates and site location in the Amendoim lake (Fig. 1C). Missing data values (in percentage) and average coverage depth by individual after filtering steps (35,638 SNPs) are also provided.

| **Sample ID** | **Site** | **Longitude** | **Latitude** | **Missing Data (%)** | **Coverage** |
| --- | --- | --- | --- | --- | --- |
| ITV20304 | Center | -50.372 | -6.399 | 0.091 | 124.75 |
| ITV20305 | Center | -50.372 | -6.399 | 0.132 | 106.86 |
| ITV20306 | Center | -50.372 | -6.399 | 0.160 | 91.43 |
| ITV20307 | Center | -50.372 | -6.399 | 0.063 | 100.18 |
| ITV20308 | Center | -50.372 | -6.399 | 0.110 | 96.83 |
| ITV20309 | Center | -50.372 | -6.399 | 0.094 | 102.07 |
| ITV20310 | North | -50.371 | -6.397 | 0.174 | 77.28 |
| ITV20311 | West | -50.372 | -6.399 | 0.094 | 142.74 |
| ITV20312 | West | -50.372 | -6.399 | 0.130 | 78.74 |
| ITV20313 | West | -50.372 | -6.399 | 0.124 | 116.26 |
| ITV20314 | West | -50.372 | -6.399 | 0.176 | 108.73 |
| ITV20316 | East | -50.371 | -6.399 | 0.135 | 98.94 |
| ITV20317 | North | -50.371 | -6.397 | 0.094 | 128.83 |
| ITV20318 | Center | -50.372 | -6.399 | 0.207 | 91.15 |
| ITV20319 | Center | -50.372 | -6.399 | 0.124 | 97.34 |
| ITV20320 | Center | -50.372 | -6.399 | 0.171 | 93.12 |
| ITV20321 | Center | -50.372 | -6.399 | 0.127 | 98.28 |
| ITV20322 | Center | -50.372 | -6.399 | 0.138 | 90.50 |
| ITV20323 | Center | -50.372 | -6.399 | 0.110 | 109.01 |
| ITV20324 | Center | -50.372 | -6.399 | 0.074 | 104.61 |
| ITV20331 | West | -50.372 | -6.399 | 0.317 | 48.36 |
| ITV20332 | West | -50.372 | -6.399 | 0.165 | 94.57 |
| ITV20333 | West | -50.372 | -6.399 | 0.127 | 88.05 |
| ITV20334 | West | -50.372 | -6.399 | 0.171 | 76.96 |
| ITV20335 | North | -50.371 | -6.397 | 0.171 | 73.39 |
| ITV20336 | North | -50.371 | -6.397 | 0.196 | 77.05 |
| ITV20337 | North | -50.371 | -6.397 | 0.204 | 65.94 |
| ITV20338 | North | -50.371 | -6.397 | 0.187 | 76.26 |
| ITV20339 | East | -50.371 | -6.399 | 0.110 | 94.04 |
| ITV20340 | East | -50.371 | -6.399 | 0.298 | 56.58 |
| ITV20341 | East | -50.371 | -6.399 | 0.174 | 77.57 |
| ITV20342 | East | -50.371 | -6.399 | 0.226 | 69.67 |
| ITV20343 | South | -50.372 | -6.402 | 0.336 | 44.23 |
| ITV20344 | South | -50.372 | -6.402 | 0.287 | 57.30 |
| ITV20345 | South | -50.372 | -6.402 | 0.201 | 69.24 |
| ITV20346 | South | -50.372 | -6.402 | 0.223 | 68.50 |
| ITV20347 | North | -50.371 | -6.397 | 0.402 | 47.86 |
| ITV20348 | North | -50.371 | -6.397 | 0.309 | 54.93 |
| ITV20349 | North | -50.371 | -6.397 | 0.232 | 78.15 |
| ITV20350 | North | -50.371 | -6.397 | 0.182 | 77.32 |
| ITV20351 | North | -50.371 | -6.397 | 0.245 | 62.31 |
| ITV20352 | North | -50.371 | -6.397 | 0.108 | 83.28 |
| ITV20353 | North | -50.371 | -6.397 | 0.243 | 58.83 |
| ITV20354 | South | -50.372 | -6.402 | 0.143 | 80.45 |
| ITV20355 | South | -50.372 | -6.402 | 0.149 | 99.02 |
| ITV20356 | South | -50.372 | -6.402 | 0.312 | 52.39 |
| ITV20357 | South | -50.372 | -6.402 | 0.198 | 68.05 |
| ITV20358 | South | -50.372 | -6.402 | 0.149 | 86.92 |
| ITV20359 | South | -50.372 | -6.402 | 0.292 | 49.90 |
| ITV20360 | East | -50.371 | -6.399 | 0.171 | 77.67 |
| ITV20361 | East | -50.371 | -6.399 | 0.389 | 50.00 |
| ITV20362 | East | -50.371 | -6.399 | 0.256 | 70.69 |
| ITV20363 | East | -50.371 | -6.399 | 0.085 | 113.89 |
| ITV20364 | East | -50.371 | -6.399 | 0.121 | 117.11 |
| ITV20365 | East | -50.371 | -6.399 | 0.113 | 85.41 |

**Table S2:** Minimum size (SIZE) in base pair (bp), number (N), total of base pair (TOTAL) of 30,000 selected contigs from the Genome Skimming approach for the samples of *I. cangae*.

| **SIZE (bp)** | **N** | **TOTAL (bp)** |
| --- | --- | --- |
| 100 | 30,000 | 16,698,786 |
| 250 | 29,957 | 16,690,814 |
| 500 | 8,082 | 9,054,021 |
| 1,000 | 3,070 | 5,677,632 |
| 2,500 | 492 | 1,903,560 |
| 5,000 | 74 | 503,032 |
| 10,000 | 4 | 53,986 |

**Table S3:** Number of SNPs retained in each filtering step to select neutral SNPs. Variant calling returned the raw SNPs, quality filters are described in Material and Methods section and linkage disequilibrium filter was performed in two step, within the same contig and between contigs, in each one the value of r^2^< 0.4. Outliers SNPs for FST values were removed using sNMF in the last step. For details see Material and Methods section.

| **Filtering Steps** | **SNPs** |
| --- | --- |
| Raw | 2,349,431 |
| Quality Filter | 71,621 |
| LD Filter (Within Contigs) | 60,377 |
| LD Filter (Between Contigs) | 36,275 |
| F_ST_ outliers (sNMF) | 35,638 |

**Table S4:** Mean, upper and lower bound of confidence intervals (C.I. 95%) for effective population size (N_e_) using linkage disequilibrium method (LD). Results from resampled datasets with different numbers of SNPs (from 5,000 to 30,000 SNPs) with no missing data are showed. Last column indicates the results using all neutral SNPs with missing data (35,638). ∞ = infinite.

| **Datasets** | **Lower** | **Mean** | **Upper** |
| --- | --- | --- | --- |
| 5,000 | 11,565.2 | 24,303.4 | ∞ |
| 10,000 | 24,832.4 | 56,707.5 | ∞ |
| 15,000 | 43,234.5 | 124,424.3 | ∞ |
| 20,000 | 45,246.2 | 92,715.8 | ∞ |
| 25,000 | 38,112.6 | 58,221.1 | 122,907.9 |
| 30,000 | 51,570.5 | 84,411.1 | 321,807.5 |
| 35,638 | 45,557.2 | 64,226.2 | 108,852.5 |

**FIGURES:**


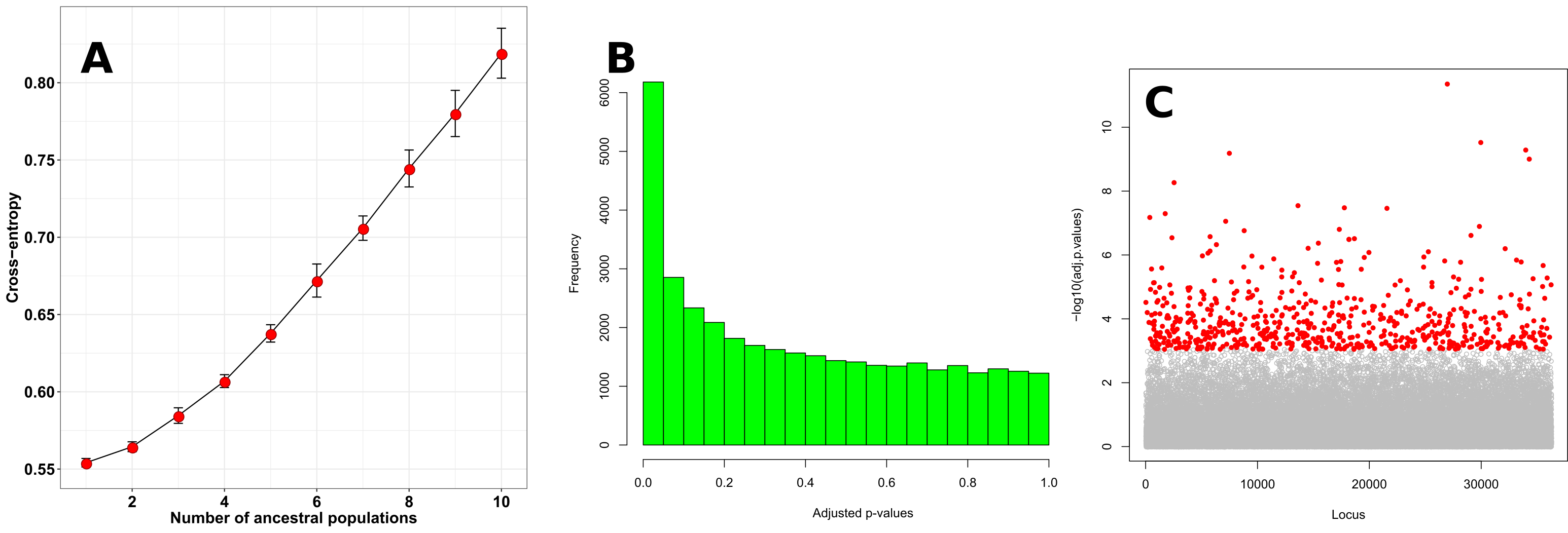


**Figure S1:** Cross-entropy for sNMF with regularization parameter α=1000 (A). We tested different values for α (10, 100, 500, 1000, 2000, 4000), all values showed the same pattern, K = 1 and K=2 with lower cross-entropy values, and we used K = 2 to select SNPs because for this analysis we need a K>= 2. Adjusted p-values with a genomic inflation factor of 0.6 to remove more SNPs (B), and Manhattan plot (C) for F_ST_ outliers, showing in red the 637 SNPs removed in this filtering step.


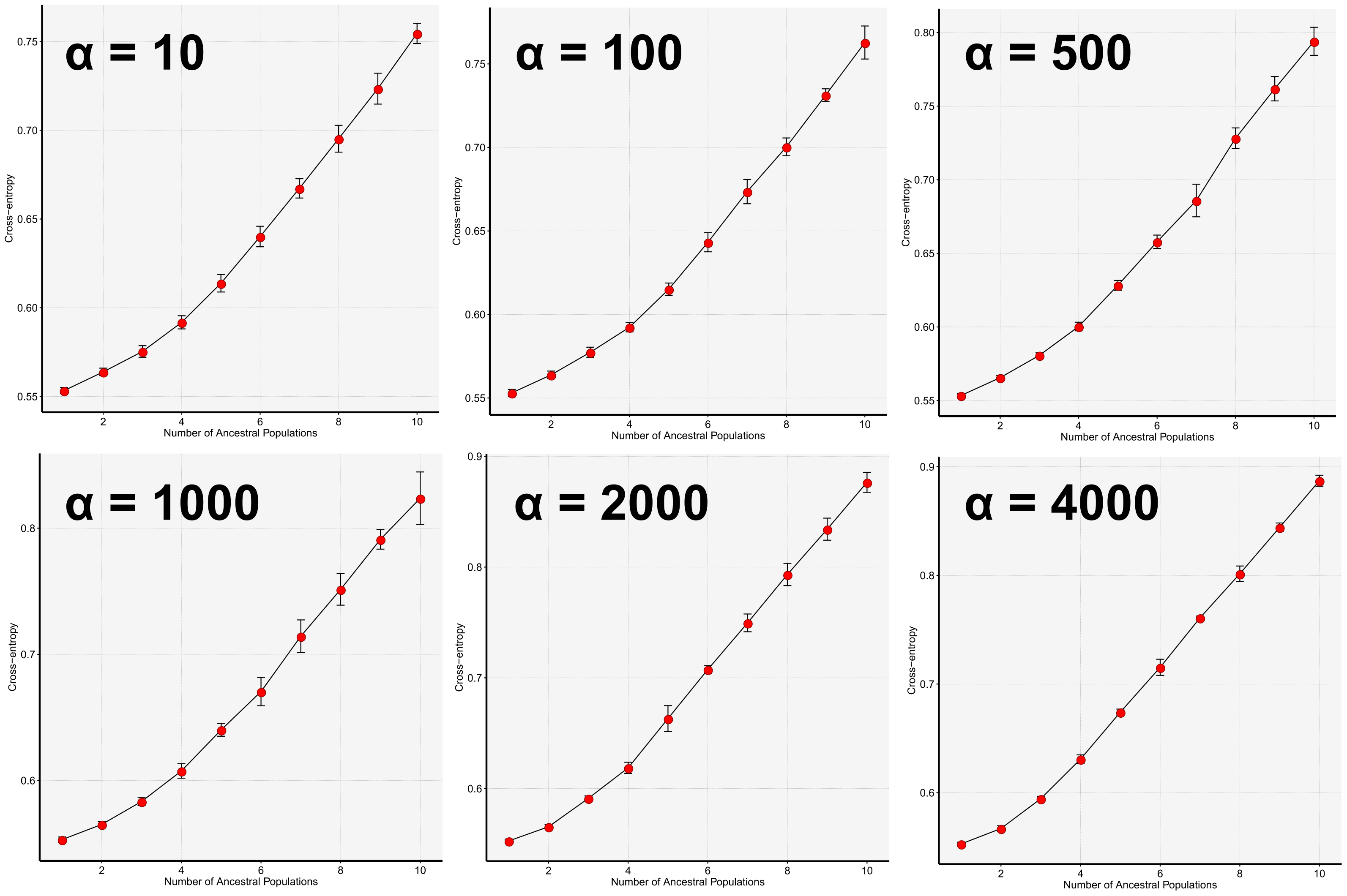


**Figure S2**: Estimates for number of ancestry populations for different values of regularization parameter (α) in sNMF.


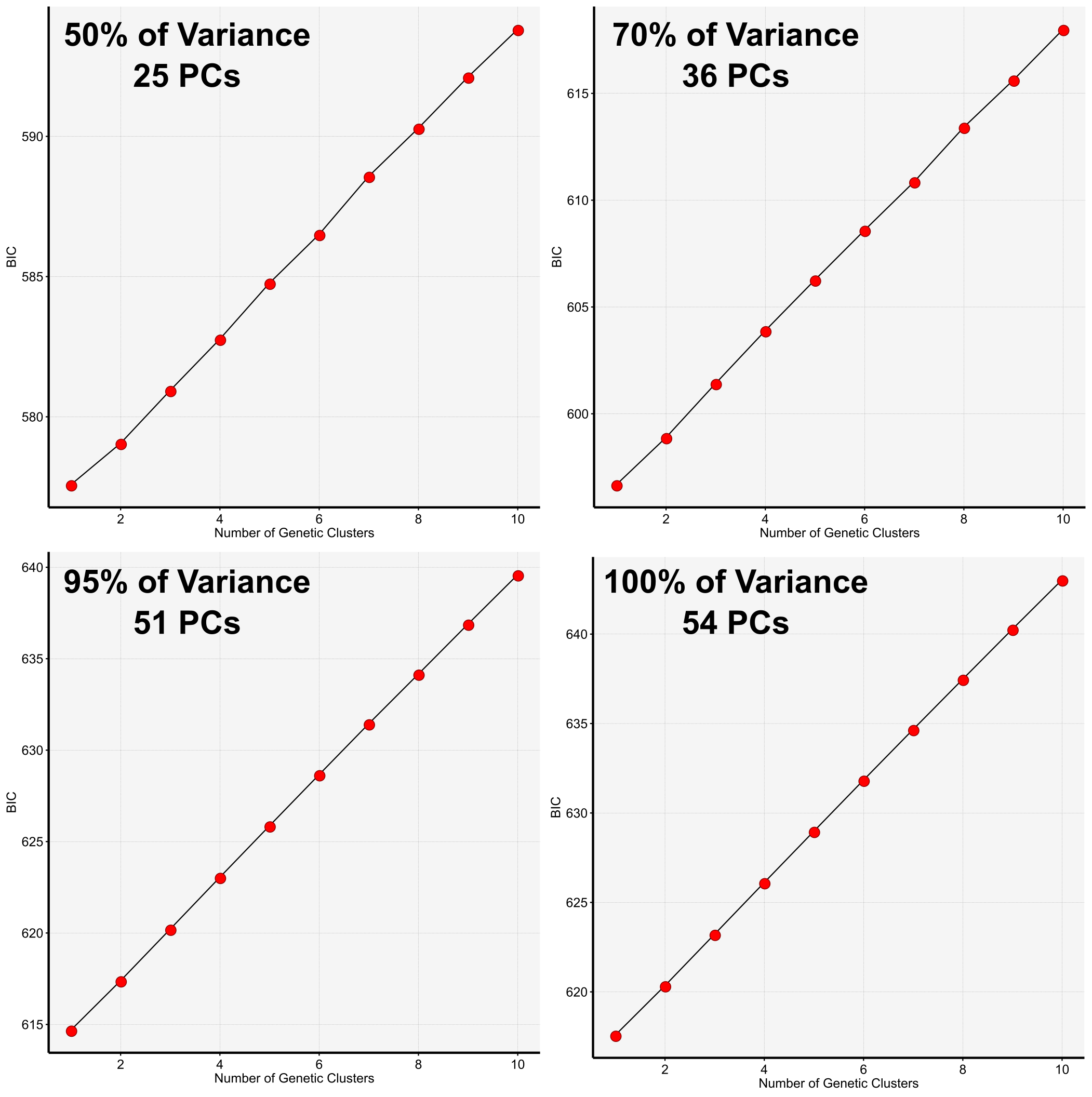


**Figure S3:** Estimates for number of genetic clusters for different numbers of principal components in DAPC.


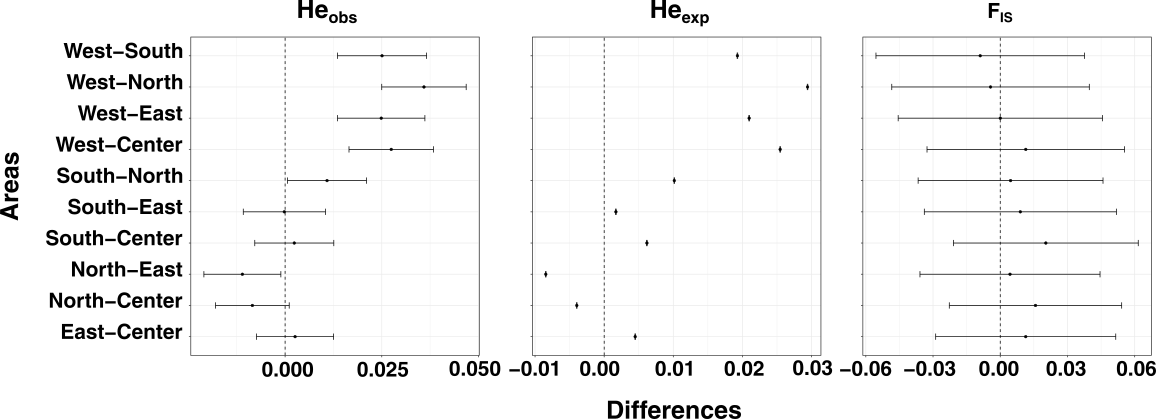


**Figure S4:** Tukey’s tests between five areas of the Amendoim lake showing significant mean differences in individual diversity metrics. Points represent the mean and bars the 95% confidence interval for each comparison. Pointed line indicate the 0, if the confidence intervals include the 0, the difference between mean is not significant. He_obs_ = observed heterozygosity; He_exp_ = expected heterozygosity; F_IS_ = inbreeding coefficient.


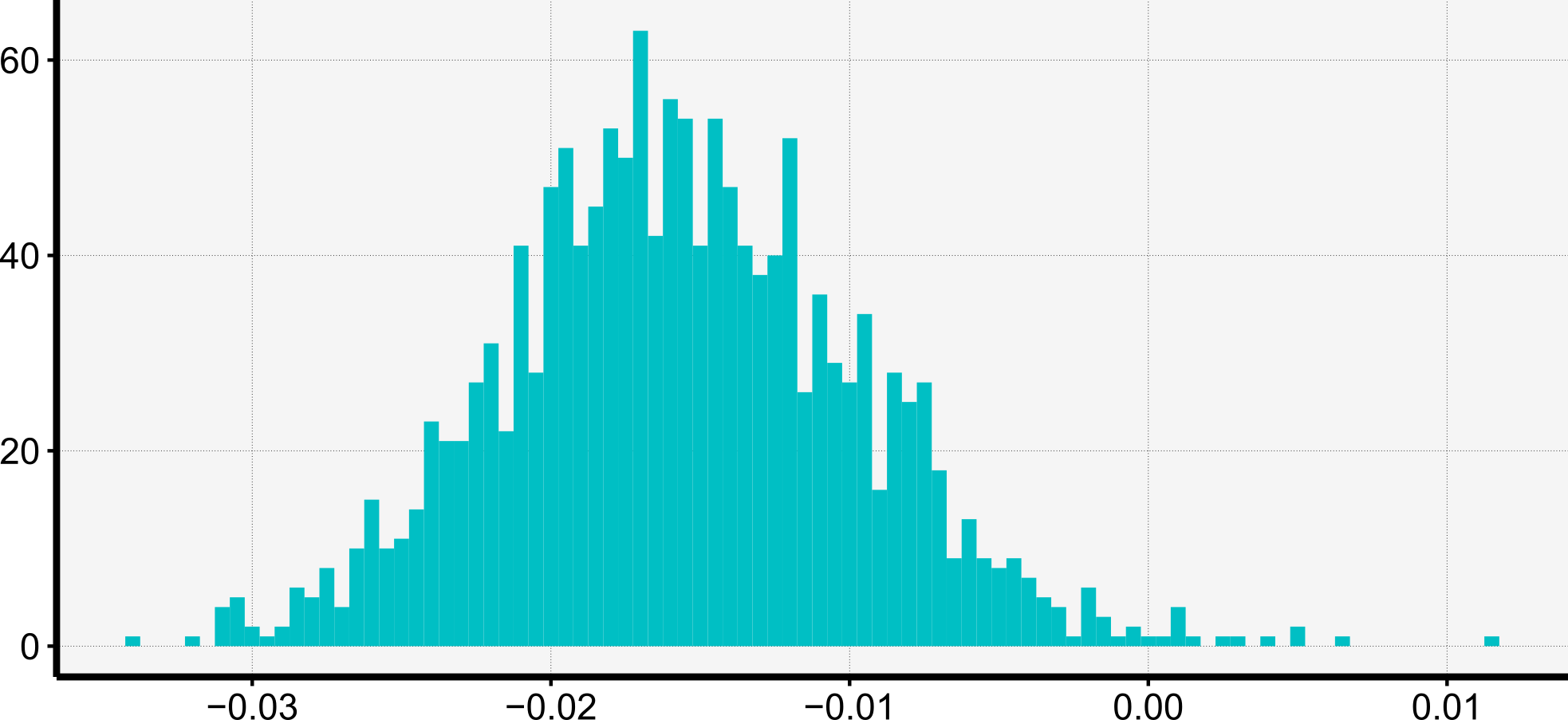


**Figure S5:** Pairwise comparisons between *Isoetes cangae* individuals for the Yang's relatedness coefficient (Rel), indicating relatedness values close to zero.
